# Systematic review comparing uretero-enteric stricture rates between open cystectomy with ileal conduit, robotic cystectomy with extra-corporeal ileal conduit and robotic cystectomy with intra corporeal ileal conduit formation

**DOI:** 10.1007/s11701-024-01850-9

**Published:** 2024-02-28

**Authors:** Daniel P. McNicholas, Omar El-Taji, Zain Siddiqui, Vishwanath Hanchanale

**Affiliations:** 1 The Royal Liverpool University Hospital, Mount Vernon St, Liverpool, L7 8YE UK; 2 University of Salford, 43 Crescent, Salford, M5 4WT UK; 3 The Christie NHS Foundation Trust, Wilmslow Rd, Manchester, M20 4BX UK

**Keywords:** Robotic cystectomy, Open cystectomy, Intracorporeal urinary diversion, Extracorporeal urinary diversion, Ureteroenteric stricture, Ileal conduit

## Abstract

Cystectomy is the gold standard treatment for muscle invasive bladder cancer. Robotic cystectomy has become increasingly popular owing to quicker post- operative recovery, less blood loss and less post-operative pain. Urinary diversion is increasingly being performed with an intracorporeal technique. Uretero-enteric strictures (UES) cause significant morbidity for patients. UES for open cystectomy is 3–10%, but the range is much wider (0–25%) for robotic surgery. We aim to perform systematic review for studies comparing all 3 techniques, to assess for ureteric stricture rates. A systematic review was conducted in accordance with the Preferred Reporting Items for Systematic Reviews and Meta-analyses (PRISMA) statement (Page et al. in BMJ 29, 2021). PubMed, Scopus and Embase databases were searched for the period January 2003 to June 2023 inclusive for relevant publications.The primary outcome was to identify ureteric stricture rates for studies comparing open cystectomy and urinary diversion, robotic cystectomy with extracorporeal urinary diversion (ECUD) and robotic cystectomy with intracorporeal urinary diversion (ICUD). Three studies were identified and included 2185 patients in total. The open operation had the lowest stricture rate (9.6%), compared to ECUD (12.4%) and ICUD (15%). ICUD had the longest time to stricture (7.55 months), ECUD (4.85 months) and the open operation (4.75 months). Open operation had the shortest operating time. The Bricker anastomoses was the most popular technique. Open surgery has the lowest rates of UES compared to both robotic operations. There is a learning curve involved with performing robotic cystectomy and urinary diversion, this may need to be considered to decide whether the technique is comparable with open cystectomy UES rates. Further research, including Randomised Control Trials (RCT), needs to be undertaken to determine the best surgical option for patients to minimise risks of UES.

**Supplementary Information:**

The online version contains supplementary material available at 10.1007/s11701-024-01850-9.

## Introduction

Radical cystectomy is the gold standard treatment for muscle invasive bladder cancer [1]. It is a unique operation, comprising of both a resection and a reconstruction element. Once the diseased bladder is removed, the reconstructive element of the operation must be performed to allow urine from the kidneys to drain. The Ileal conduit is the most popular urinary diversion but orthotopic neobladder formation is increasing in popularity [2, 3]. The first robotic assisted radical cystectomy was by Menon et al. [4]. The robotic technique has been described as having many advantages for the patient such as less blood loss, shorter length of stay and decreased post- operative pain, whilst also being ergonomically advantageous for the surgeon [5, 6]. A meta-analysis has suggested that further high- level evidence is needed to show robotic cystectomy is superior to open surgery, but current evidence shows they have similar oncological outcomes and similar major complication rates [7].

Uretero-enteric stricture (UES) is a complication which causes significant morbidity for patients, and places burden on healthcare systems. It often leads to further unplanned hospital visits with issues such as pain, infection and obstructive uropathy [8]. There may be a need for further procedures or interventions to be performed such as nephrostomy, ureteric stent insertion or re-implantation of the ureter. Incidence of UES in open cystectomy is relatively low (3–10%) [9, 10]. However, there is much heterogeneity in the literature regarding these results with stricture rates for robotic surgery, ranging from 0 to 25% in most recent studies [11–13].

The aetiology of UES is thought to be multifactorial. There are several hypotheses including the type of anastomoses, surgical technique, patient factors, and surgical experience. One theory is that the type of anastomosis has a role to play in the development of uretero-enteric stricture. The two most common anastomotic techniques are the Bricker anastomoses and the Wallace anastomosis. The Bricker anastomotic technique was described in the 1950’s and involves anastomosing each ureter separately into a segment of bowel, with the distal end of the segment brought to the skin [14]. The Wallace anastomotic technique, described in the 1960’s, involves spatulating the distal ends of both ureters and anastomosing their medial walls. The free edges are then anastomosed to the proximal end of the bowel segment. The distal end of the bowel is brought to the skin as in the Bricker technique [15]. However, a meta-analysis has found no significant difference between the techniques and their respective rates of uretero-enteric stricture formation [16].

Other hypothesis for UES formation includes surgical technique, this can result in excessive disruption of the adventitia on the ureters which causes ischaemia. It has also been suggested that too much tension on the anastomosis, trauma to the ureter from poor handling and inflammation can all contribute to the formation of UES [17]. There are many other factors which may contribute to the development of UES. Patient factors such as obesity, previous abdominal surgery or chemoradiotherapy have all been associated with UES, as well as peri-operative factors such as urine leak or urine infection [12, 18, 19]. It has been suggested that a robotic approach to cystectomy can increase the incidence of UES formation compared to open operations [12]. However, there is much heterogeneity in the literature regarding these results with stricture rates for robotic surgery, ranging from 0 to 25% in most recent studies [11–13]. Robotic surgery is in its infancy, and some studies have demonstrated a learning curve for surgeons. It is particularly interesting and encouraging to note that stricture rates have decreased over time in those studies looking at this learning curve [12, 20].

Robotic urinary diversion was initially performed using an extracorporeal technique as it is less technically demanding and is thought to be quicker. A recent review of the International Robotic Cystectomy Consortium (IRCC) database showed that 82% of over 900 patients received an extracorporeal urinary diversion [21]. However, the intracorporeal technique is gaining popularity. This is due to perceived benefits such as less pain, less intra-operative blood loss, less evaporation and smaller surgical incisions when compared with the extracorporeal technique [22]. It is unclear which technique is best to avoid UES, and there are not many studies comparing these techniques and their outcomes.

In this systematic review, we aim to investigate and identify if there is a difference in uretero-enteric stricture rates between open cystectomy with ileal conduit, robotic cystectomy with extra-corporeal ileal conduit and robotic cystectomy with intra corporeal ileal conduit formation.

## Methods

### Search strategy

The review was prospectively registered (PROSPERO ID: CRD42023428050) with a review question, search strategy, inclusion/exclusion criteria are detailed below. A systematic review was conducted in accordance with the Preferred Reporting Items for Systematic Reviews and Meta-analyses (PRISMA) statement [23]. PubMed, Scopus and Embase databases were searched for the period January 2003 to June 2023 inclusive for relevant publications.

#### Review question

To compare the uretero-enteric stricture rates between open cystectomy and urinary diversion, robotic cystectomy and intracorporeal urinary diversion (ICUD) and robotic cystectomy and extracorporeal urinary diversion (ECUD).

#### Search strategy

Patients are required to have a cystectomy. The paper must report outcomes from patients having either a robotic cystectomy, including intracorporeal and extracorporeal techniques, or an open cystectomy. Using [cystectomy] as a search term will identify all these papers.

The outcome measure we are searching for is uretero-ileal strictures. These happen in patients who have had a urinary diversion performed as part of their cystectomy. In forming a urinary diversion, a segment of bowel and ureter are connected. This anastomosis is described mostly as uretero-enteric or uretero-ileal anastomosis. We will search terms to include these patients [Urinary diversion OR uretero-ileal anastomosis OR uretero-enteric anastamosis].

The two most common surgical techniques to perform the anastomosis is the Bricker, and the Wallace techniques. We will include [Bricker AND/OR Wallace] in our search strategy.

We want to identify patients who have strictures to their ureter or the anastomosis after having their surgery. By using the search term [Stricture], we will identify all these papers.

#### Search terms

(cystectomy) AND ((Bricker AND/OR Wallace) OR urinary diversion OR uretero ileal anastomosis) AND (stricture).

### Eligibility criteria

#### Study design

No restrictions are being placed on the population included in the study.

Included:

- Primary papers (including randomized control trials, prospective and retrospective case series, from both single centre or multiple centre studies) reporting uretero-enteric strictures as an outcome after open and robotic cystectomy, with both intracorporeal and extracorporeal urinary diversion reported.
- Benign and malignant indications.

Excluded:

- Review articles.
- If ureteric strictures not reported in outcomes.
- Papers reporting revision surgeries for ureteric strictures.
- Papers published in 2002 or earlier (The first robotic cystectomy was described in 2003).

### Intervention

Open cystectomy and urinary diversion (open), robotic cystectomy with extracorporeal urinary diversion (ECUD) and robotic cystectomy with intracorporeal diversion (ICUD).

### Data extraction

Two reviewers DM and OE independently reviewed the literature for primary studies looking at ureteric stricture rates for patients undergoing open cystectomy with ileal conduit, robotic cystectomy with intracorporeal ileal conduit and robotic cystectomy with extracorporeal ileal conduits. Papers identified were reviewed and suitable papers meeting the inclusion criteria were included. Any disagreements between individual judgements were discussed with the study supervisor VH who is very experienced in academia and a robotic surgeon with > 10 years’ experience.

The information was recorded on a Microsoft excel database.

The following data was extracted and recorded:

- Primary studies reporting ureteric stricture rates for patients undergoing open, robotic intracorporeal and robotic extracorporeal urinary diversion.
- Patient sex, age and demographics.
- Number of patients undergoing 1 open, 2 robotic intracorporeal and 3 robotic extracorporeal urinary diversion.
- The type of anastomosis performed for each patient (eg. Bricker or Wallace technique).
- The type of urinary diversion performed (ileal conduit, neo-bladder).
- Author names.
- Journal and year of publication.
- Study type.
- Enrolment dates.
- Length of follow up.
- Total number of patients.
- Total number of ureters.
- History of neo-adjuvant radiotherapy and/or chemotherapy.
- Indication for ureteroenteric anastomosis.
- Imaging modality for diagnosing ureteroenteric strictures.
- The number of uretero-enteric strictures for:
  1. Open.
  2. ICUD.
  3. ECUD.
- Which side/sides the uretero-enteric strictures have occurred on.
- Time to stricture formation.
- Length of follow up.

One reviewer DM independently extracted the data and this was checked by 2nd reviewer ZS. Studies with incomplete data for primary outcomes were not included in the study as per inclusion criteria. Studies that didn’t include data for secondary outcomes were included in the study and it has been acknowledged that data is missing in this case.

### Outcome measures

#### Primary outcome

Uretero-enteric stricture rates following cystectomy and urinary diversion formation for open, ECUD and ICUD techniques.

#### Secondary outcomes

- Side of the ureteric stricture (left vs right) and identify if there are any predisposing factors if one side is more affected.
- Time (in months) to stricture.
- Rate of intervention.
- Type of intervention (endoscopic/percutaneous/surgical).
- Length of surgery (in minutes).
- Impact of surgeon volume on stricture rate.
- Stricture rate for Bricker anastomoses.
- Stricture rate for Wallace anastomoses.

### Risk of bias assessment

The Newcastle Ottawa Scale (NOS) was used to assess the quality of the studies in this systematic review, with scores ranging from 0 to 9 points. The NOS is a review tool for evaluating risk of bias in observational studies. The scale consists of four domains of risk of bias assessment; (i) selection bias; (ii) performance bias; (iii) detection bias and; (iv) information bias [24].

### Data reporting and statistical analysis

Data are presented as average/mean. Time to stricture was presented in months. Operative length was presented in minutes. Meta- analysis was not performed due to the small number of studies identified comparing all three techniques and the absence of a randomised control trial.

## Results

### Eligible studies

**Fig. 1:**
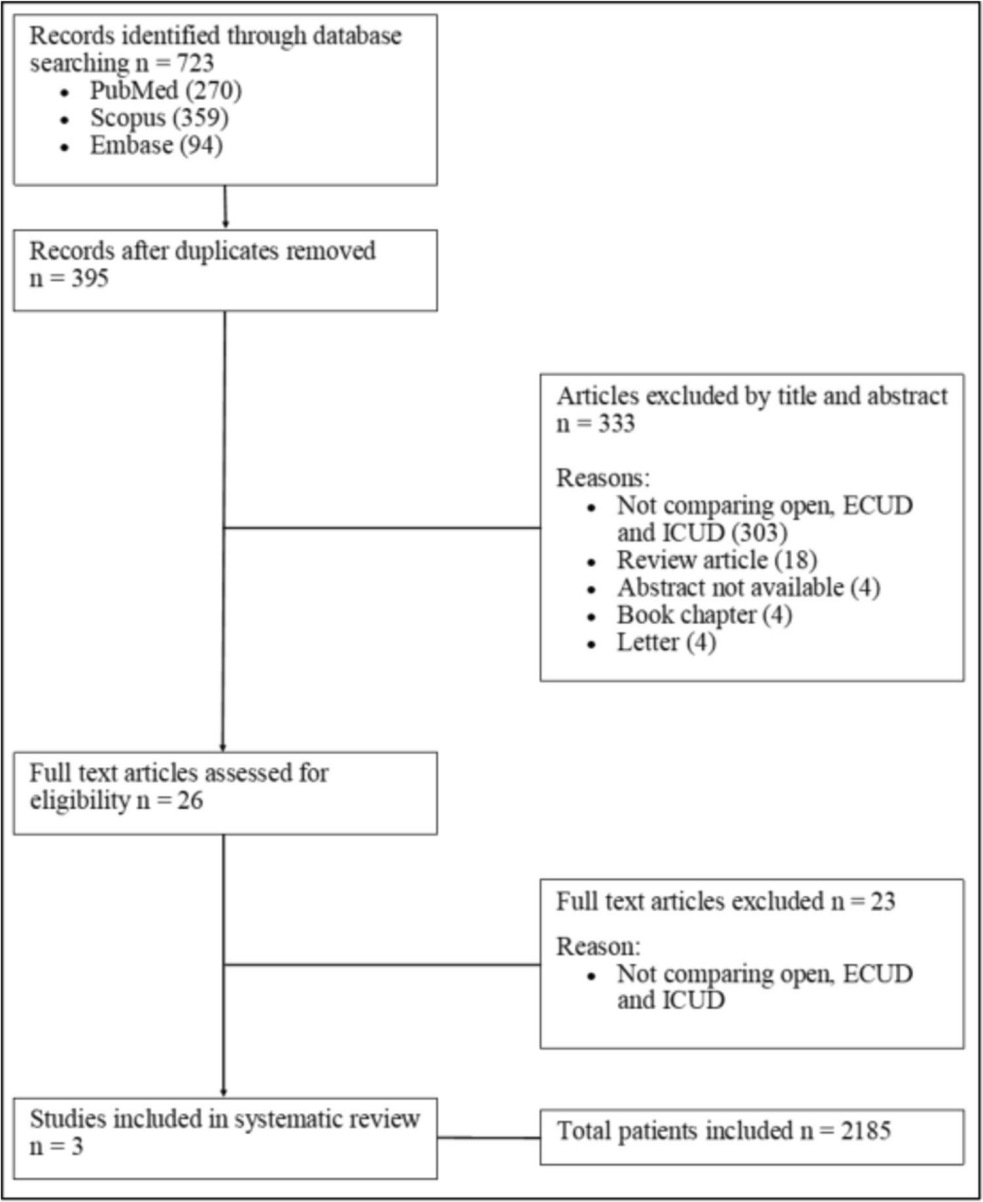
PRISMA (Preferred Reporting Items for Systematic Reviews and Meta-Analysis) diagram of the studies identified in the systematic review [23]

Three studies were identified which compared outcomes for open cystectomy and urinary diversion, robotic assisted cystectomy with extracorporeal urinary diversion and robotic assisted cystectomy with intracorporeal urinary diversion. A total of 2185 patients were included in the study. All three studies performed retrospective analysis of prospectively maintained databases for the patients in their institutions (Table 1). The initial search identified 723 articles and 26 full text articles were reviewed and assessed for eligibility, 23 of which were excluded (Fig. 1). All three studies were in English and were published in the last 3 years. The cohort of patients are reflective of modern practice, and all underwent cystectomy and urinary diversion for bladder cancer. The Newcastle Ottowa Scale was used to assess for risk of bias (Table 2) and study quality (Table 3) [24]. All three studies included within the paper were classified as good quality.

**Table 1:** Summary of study characteristics [18, 19, 25]

Year published	Country	Journal	Author names	Study type	Enrolment dates	Total patients	OPEN patients	ECUD patients	ICUD patients
2020	USA	Laparoscopy and robotics	Kyle J Ericson et al.	Single centre retrospective review	January 2011 to May 2018	968	279	382	307
2020	USA	Urologic Oncology	Zaem Lone et al.	Single centre retrospective review	2010 to 2018	644	193	260	191
2021	USA	International Journal of Urology	Kassem S Faraj et al.	Single centre retrospective review	01/01/2007 to 01/01/2018	573	337	197	39
				Overall total patients		2185	809	839	537

**Table 2:** Newcastle-Ottawa scale risk of bias assessment adapted for Cohort Studies: A study can receive a maximum of one star for each numbered item in the Selection and Outcome categories. A maximum of two stars can be given for comparability

Study	S				C	O			Total
	Representativeness	Selection exposed to cohort	Ascertainment	Result not present at start of study	Comparability for confounders	Assessment of outcome	Follow up duration	Adequacy of follow up	
Ericson et al. [19]	*	–	*	*	*	*	*	*	7
Lone et al. [25]	*	–	*	*	*	*	*	*	7
Faraj et al. [18]	*	–	*	*	*	*	*	*	7

A maximum of two stars can be given for comparability [24]

**Table 3:** Newcastle–Ottawa scale quality of evidence

Study	Total Newcastle- Ottowa Score	Study quality
Ericson et al. 2020	7	Good
Lone et al. 2020	7	Good
Faraj et al. 2021	7	Good

Good quality: 3 or 4 stars in selection domain AND 1 or 2 stars in comparability domain AND 2 or 3 stars in outcome/exposure domain. Fair quality: 2 stars in selection domain AND 1 or 2 stars in comparability domain AND 2 or 3 stars in outcome/exposure domain. Poor quality: 0 or 1 star in selection domain OR 0 stars in comparability domain OR 0 or 1 stars in outcome/exposure domain [24] (Tables 4, 5)

**Table 4:** Results for the individual surgical procedures from each study (NR-not recorded) [18, 19, 25]

List	Open			ECUD			ICUD		
	Ericson et al.	Lone et al.	Faraj et al.	Ericson et al.	Lone et al.	Faraj et al.	Ericson et al.	Lone et al.	Faraj et al.
Patients	279	193	337	382	260	839	307	191	39
Age	69.4	67	71	69.7	67	71	68.9	67	76
Male	233	164	NR	319	223	NR	238	147	NR
Body mass index	27.5	28.6	28.1	28.1	28.7	28	27.5	28.6	27
Charlseton co-morbidity index	3	3.3	3.1	3	3.4	3	3	3.4	5
Neo-adjuvant treatment	85	66	159	141	98	99	110	69	19
Ileal conduit	210	137	295	273	172	164	261	152	37
Neo bladder	41	34	42	44	38	33	40	39	2
Ileal pouch	27	22	0	64	50	0	6	0	0
Bricker	279	NR	249	382	NR	138	307	NR	15
Wallace	0	NR	88	0	NR	58	0	NR	24
Operation duration (minutes)	NR	344	300	NR	443	367	NR	412	359
UES	26	25	27	43	42	19	40	36	1
Left	14	NR	NR	19	NR	NR	22	NR	NR
Right	9	NR	NR	13	NR	NR	12	NR	NR
Bilateral	1	NR	NR	11	NR	NR	6	NR	NR
Interventions	24	NR	NR	36	NR	NR	37	NR	NR
Time to stricture (months)	4.5	NR	5	4.7	NR	5	5.1	NR	10
Length of follow-up (months)	22	NR	55	10.6	NR	70	18.3	NR	89.3

**Table 5:** Combined results for the individual surgical procedures—open, robotic extracorporeal urinary diversion and robotic intracorporeal urinary diversion

	Open	ECUD	ICUD
List	Number (%)	Number (%)	Number (%)
Patients	809	839	537
Age	69.1	69.2	70.6
Male	397 (49%)	543 (64%)	385 (72%)
Body mass index	28	28	27.7
Charlseton co-morbidity index	3.1	3.1	3.8
Neo-adjuvant treatment	310 (38%)	338 (40%)	198 (37%)
Ileal conduit	642 (79%)	609 (73%)	450 (84%)
Neo bladder	117 (14%)	115 (14%)	81 (15%)
Ileal pouch	49 (6%)	114 (14%)	6 (1%)
Bricker	528	520	322
Wallace	88	58	24
Operation duration (minutes)	321.75	405.2	385.7
UES	78 (9.6%)	104 (12.4%)	77 (14%)
Left	14	19	22
Right	9	13	12
Bilateral	1	11	6
Interventions	24	36	37
Time to stricture (months)	4.75	4.85	7.55
Length of follow-up (months)	38.5	40.3	44.65

The study periods are listed in Table 1. Ericson’s paper had 968, Lone’s 644 and Faraj’s paper 573 patients in total [18, 19, 25]. Ileal conduit was the most popular urinary diversion technique (n = 1701), other urinary diversion types performed included neo-bladder, ileal pouch and continent conduit. The Bricker anastomosis was the most popular technique recorded (n = 1370). There were 259 (11.85%) strictures described in total. A definition for ureteric stricture was described in two studies. Ericson described ureteric stricture as hydronephrosis confirmed by functional imaging, or unexplained hydronephrosis with loss of renal parenchyma [19]. Faraj describes stricture as radiological hydronephrosis and obstruction at the level of the anastomosis [18]. The most common acute interventions were nephrostomy or JJ stent (n = 118) Faraj used nephrostomy for 76%, re-implantation for 15% and endoscopic procedures with general anaesthesia for 6%. Ericson describes chronic percutaneous/ trans-stomal drainage for 64%, short term drainage for 7% and ureteral re-implantation for 18% of patients respectively. In all three papers, 27 patients went on to have ureteric re-implantations (n = 27). Most patients in the study had left sided strictures (n = 79).

### Open group

There were 809 patients included in this category. The average age was 69.1 years. Most patients were male in the two papers that described sex (n = 397). The average Body Mass Index (BMI) was 28. The Charleston co-morbidity index (CCI) was used in each study to categorise co-morbidities, the average value was 3.1. Over one-third of patients underwent neo-adjuvant treatment (n = 310).

Most patients had an ileal conduit urinary diversion performed (n = 642). Neobladder was performed for 117 patients and Ileal pouch in 49 patients. Two papers described operative duration (average n = 321.75 min). Two papers described anastomotic technique. Ericson only used Bricker anastomosis (n = 279), while Faraj used both Bricker (n = 249) and Wallace (n = 88).

Benign strictures were recorded in 78 patients after open urinary diversion (9.6%). Only Ericson described laterality of the stricture (left-14, right-9, bilateral 1, transplant ureter-1.) and the number of interventions (n = 24). Both Ericson and Faraj described the time to stricture formation (average 4.75 months) and length of follow up (22 months and 55 months respectively).

### Extracorporeal group

A total of 839 patients were included. Most patients were male (n = 542, 65%) and the average age was 69.2 years. All three studies reported BMI, the average was 28. All three studies recorded the Charleston co-morbidity index (CCI), the average value was 3.1. All three studies recorded patients undergoing neo-adjuvant treatment (n = 338).

Ileal conduit was the most popular urinary diversion (n = 609). Neo-Bladder and Ileal pouch formation was recorded for 115 and 114 patients respectively. Both Faraj and Lone’s papers described operative length (average = 405.2 min). Two papers described their anastomotic technique. Ericson only performed Bricker anastomosis (n = 382), while Faraj describes Bricker for 138 patients and Wallace for 58 patients.

All three studies recorded the number of strictures identified. There were 104 benign anastomotic strictures recorded in total. Only Ericson described laterality of the stricture (left-19, right-13, bilateral 11) and the number of interventions (n = 36). Ericson and Faraj described the time to stricture formation (average 4.85 months), and length of follow up (10.6 and 70 months respectively).

### Intracorporeal

A total of 507 patients were included, of which 385 were male. All three studies recorded BMI and CCI, the mean values were 27.7 and 3.8 respectively. A total of 198 patients had neo-adjuvant treatment.

In total, 450 ileal conduit urinary diversion procedures were performed. Neo-Bladder and Ileal pouch formation accounted for 81 and 6 patients respectively. The average operating time from the two papers which described this was 385.7 min. Ericson performed 307 Bricker anastomosis while Faraj performed 15 Bricker and 24 Wallace intracorporeal uretero-enteric anastomosis.

There were 77 benign strictures identified in the 3 studies. Only Ericson describes the laterality of the strictures (left 22, right 12, bilateral 6) and interventions (n = 37). Both Ericson and Faraj describe the time to stricture formation (average 7.55 months) and length of follow- up, 18.3 and 71 months respectively.

### Comparison of techniques and outcomes

#### Strictures

All three studies compared stricture rates. The open operation had the lowest stricture rate (9.6%), compared to ECUD (12.4%) and ICUD (15%).

#### Time to stricture

This was described by Ericson’s and Faraj’s papers. The ICUD technique had the longest time to stricture (7.55 months), ECUD was 4.85 months and patients undergoing the open operation got strictures sooner than both other techniques (4.75 months).

#### Length of surgery

Lone’s and Faraj’s papers compared duration of surgery. Open was the fastest (321.75 min), ECUD was the longest (405.2 min), while ICUD took on average 385.7 min.

#### Anastomotic technique

Both Ericson and Faraj described whether they used Bricker or Wallace techniques. The Bricker technique was most popular overall as already described. This was used in atleast 1370 (63%) of the total papers’ patients. Lone did not specify about technique in their 644 patients.

For the open technique, 528 (65%) patients had Bricker technique used, 88 had Wallace and 193 were unknown. The ECUD had 520 (62%) patients undergoing a Bricker anastomoses, 58 had a Wallace performed and 261 were unknown. The ICUD had 322 (64%) who underwent Bricker procedure, 24 had a Wallace and 161 were unknown.

#### Demographics and co-morbidities

These were similar between all three cohorts. Patients had a similar age in all three cohorts (open-69.1 years, ECUD-69.2 years, ICUD-70.6 years). ICUD patients were mostly male (72%), similarly with ECUD (64%), and open surgical patients were 49% male. Patient’s having an open and ECUD operations had an average BMI of 28 and ICUD patient’s average BMI was 27.7. The Charelston Co-Morbidity Index for open and ECUD was 3.1, for ICUD it was 3.8. The number of patients undergoing neo-adjuvant treatment was 310 (38%) for those having the open procedure, 338 (40%) for the ECUD group, and 198 (39%) for those in the ICUD group.

### The learning curve

Ericson’s paper describes their learning curve with robotic cystectomy. At the beginning of their study period in 2011, 4 cystectomies were approached using ICUD, 51 ECUD and 63 were open. In 2014 the cumulative numbers were 132, 141 and 206 respectively. By the end of the study in 2018 the cumulative numbers had shifted further towards robotic cases with 307 (ICUD), 382 (ECUD) and 279 (open) respectively. They reviewed surgeon experience in the ICUD cohort of patients. In total five surgeons performed ICUD. Three surgeon’s performed 182, 71, and 34 cases each. Two further surgeons performed < 25 cases each. Stricture rates decreased as surgeon experience (measured by case volume) went up. Stricture rate prior to 75 cases was 17.5%, and decreased to 4.9% after 75 cases. In this stud notably only one surgeon performed over 75 cases. This paper noted that on multivariate analysis, higher case numbers were independently associated with reduced risk of stricture. The other two papers in this review did not include results for surgeon experience.

## Discussion

Robotic cystectomy is becoming increasingly popular and is rapidly becoming the operative approach of choice for surgeons operating on bladder cancer. While open surgery is currently the gold standard surgical approach for radical cystectomy, much more conclusive evidence is required before robotic surgery can be proven to be superior. The RAZOR trial is the only Randomised Control Trial (RCT) comparing open and robotic cystectomy, however it did not describe ICUD and ECUD uretero-enteric stricture rates [26]. To our knowledge, our systematic review comprising of 2185 patients, is the first to compare UES outcomes between open, ECUD and ICUD robotic techniques.

There have been widely varied reports of UES rates associated with robotic ICUD, Reesink et al. described 25% and Ahmed et al. described 16% [12, 27]. These papers have variable sample sizes 87 and 400 respectively. In one of the papers included in our review, UES rate for was only 2.5% but in a small cohort of patients (n = 39) [18]. There is similar variability in the reporting of UES for ECUD procedures. No strictures were reported in a small study population of 56 patients [11]. An earlier study describes 12% stricture rate for ECUD procedures in a cohort of 103 patients [28]. Open cystectomy has well described UES rates (3–10%) [5, 6, 27]. A RCT has shown UES for open (7%) and robot (9%) but did not differentiate between ECUD and ICUD in the robotic cohort [26]. In our review, all papers described their stricture rates for each procedure. The mean UES rate were calculated for open (9.6%), ECUD (12.4%) and ICUD (15%). These figures are in line with the current literature available, suggesting open has the lowest UES rates while ICUD has the highest. Interestingly, regarding robotic ICUD, one paper in our review identified that the risk of UES prior to a surgeon’s 75th case was 17.5% but this figure dropped to 4.9% thereafter [19]. Another paper in our study overall had very low ICUD UES rates (2.6%). They commented that most of their robot procedures, including all their ICUD procedures were performed by one surgeon who has considerable experience in robotic surgery [18]. This highlights an interesting point regarding the significance of the learning curve for robotic cystectomy, and the resultant UES rates.

The learning curve was defined by the IRCC in 2015 as 20–30 operative cases [22]. In the first 30 cases it is expected that the surgeon will reach specific targets, including blood loss and operative time. Once 30 cases have been reached, the surgeon is considered experienced and has completed the initial learning curve [22]. One study looking at the learning curve for surgeons noted that the incidence of UES decreased from 29% after the first 20 cases, to 9% for the final 22 cases in a series of 62 consecutive robotic ICUD [20]. Another similarly showed that UES rate was 35% for the first 20 patients but decreased to 15% for the last 20 patients in their study who had a robotic ICUD performed [12]. These further strengthen the argument that UES rates are decreased as surgeon experience increases.

It is described in the literature that UES rates increase with time post operatively. One study describes UES rates of 13% in the 1st year post-operatively, but this rate increased to 19% at 5 years [13]. Similarly, another paper had a UES rate of 13% at a median of 5 months and 19% at 5 years [13]. It has been suggested there is an under reporting of UES in the literature due to wide ranging follow up schedules [13]. The median time to diagnosis of stricture has been described as 4–18 months [28] and as 3 months for ECUD, but 5 months for ICUD [27]. In our review, strictures development ranged from 4.75 months for open cystectomy and 7.55 months for ICUD.

There is evidence of wide-ranging following schedules in this review. The follow up schedules range from 22 to 55 months for open cystectomy, 11–70 months for ECUD and 18–71 months for ICUD, as described in our results section. The European Association of Urology guidelines (2023) recommend a CT scan every 6 months up until and including the 3rd year, with annual imaging thereafter [29]. It doesn’t however give a timeframe for this intensive follow up. It is acknowledged that there is a wide variation in follow up requirements, depending on the risk profile of the individual patient’s disease. More investigation needs to be done to create an agreed follow up schedule, including required imaging, for patients post cystectomy. This will require risk stratifying patients according to their disease. This will help add some uniformity in future studies assessing the incidence of UES and time to stricture formation.

The average operating time for open, ECUD and ICUD in our review were 322, 405, 386 min respectively. Both our ECUD and ICUD figures have reached the recommended goals for the learning curve stage for surgeon’s but have not reached the target set for the experienced surgeon [22]. The reason for this is likely multi-factorial including- multiple different surgeons performing the operation who may be at different stages of experience, as well as variation in the complexities of the cases. One paper in our study did not describe how many surgeons have performed the operations in their study or commented on their experience [25]. However, one other paper commented that the majority if their robot cases, including all ICUD cases, were done by a single surgeon [18]. This may explain the shorter operating time for the robotic cases in their study. The average operating time for open cystectomy in our review was 322 min. This is shorter than both robotic techniques and this is already well known and was shown in another systematic review. They also concluded that operative times are dependent on the surgeon and their experience [22].

Both Bricker and Wallace anastomoses are well described for urinary diversion. Ericson et al. only used a Bricker anastomoses [19], while Faraj et al. used both Bricker and Wallace anastomoses [18]. Bricker was the most common technique used, with over half of all patients involved in the study having a Bricker anastomoses. In both the open and the ECUD groups, most patients received a Bricker anastomoses, however in the ICUD group by Faraj et al. there were more Wallce (24) than Bricker (15) anastomoses performed [18]. The decision about which technique to use was at the discretion of the surgeon. It has previously been shown that there is no difference between either technique regarding risk of UES. None of the papers in our review describe their UES stricture rates in relation to the type of anastomoses deployed [16]. Historically it was felt that Wallace anastomoses was quicker and easier to perform. There were concerns about bilateral ureteric obstruction and contralateral upper tract seeding in some cases, but there was thought to be a decreased risk of UES formation [30]. This may explain why the Wallace technique was more popular for ICUD.

Only one study described the side of stricture in our study [19]. The left sided stricture rates for open (56%), ECUD (44%) and ICUD (55%) are in line with previous descriptions in the literature. UES is more common on the left side, and this has been widely reported [12, 13, 19, 27]. The conduit is usually placed on the right side of the abdomen, therefor the left ureter requires more dissection to mobilise it so it can be tunnelled under the mesentery to the right-hand side. This requires more handling of the ureter, increasing the risk of traumatic handling and skeletisation [31]. This may explain the higher rate of left sided UES. Furthermore, stretching of the ureter while forming the conduit out of the abdomen is also thought to be a risk factor for UES formation. It is hypothesized that ICUD will have less UES due to less stretch being placed on the ureter to perform the anastomoses outside of the abdomen [32]. However, this has not been shown in our result. It is worth noting that new techniques are emerging, which may help to improve the UES rates for ICUD procedures. Indo Cyanine Green (ICG) is a substance which can be used to help identify the vascular supply of the distal ureter when viewed using Near Infrared Fluorescence (NIRF) [33]. This technique removes the subjective assessment of the vascularity of the distal ureter, non-enhancing tissue is excised and a well perfused anastomoses formed. Ahmadi et al. describe a 0% stricture rate in their case series of 47 consecutive patients where ICG technology was used [33]. Another study has shown stricture rate of 7.5% prior to adopting ICG, and this improved to 0% once ICG was used [34]. These results are very promising, however further research will need to be done on a larger scale to confirm the suggested beneficial outcomes for patients.

There are several limitations to our study. We had strict inclusion/exclusion criteria for this study, which limited the number of studies that could be involved and resulted in us not performing meta-analysis. In the future it would be useful to broaden the inclusion criteria to include studies comparing two of the three techniques in this study (open vs ECUD, open vs ICUD, ECUD vs ICUD), which would allow us to pool and compare the respective data for much larger patient numbers and give us good data for a meta-analysis. There were no RCT’s included in our study population as none have been performed which compare UES rates for open, ECUD and ICUD surgeries. Within our included studies, although all three reported UES rates for the respective surgeries, there was incomplete data regarding surgeon experience, intervention for UES, laterality of stricture formation, time to stricture formation, length of follow up and operative duration. Regarding surgical technique for Bricker or Wallace anastomosis, data was incomplete as one study did not record which technique was used and none of the studies described their UES rates in relation to the technique used. Statistical analysis was not used due to the large amount of incomplete data for our secondary outcomes.

In conclusion, we describe a comparison of uretero-enteric stricture rates for open, ECUD and ICUD robotic techniques. This review has the largest cohort of patients available in the current literature and we have shown similar UES rates to those that are previously described. It has been highlighted in our review about the significance of the learning curve for robotic surgery. This should be considered when describing the results for complications such as UES because inevitably there will be higher than usual rates of complications, longer operating times, more blood loss in the first 30 cases as already described. Many prior studies only report small patient populations, therefor the 30 cases of the learning curve may represent a high proportion of this. New techniques for robotic surgery such as Indo Cyanine Green may also help to improve outcomes and decrease stricture rates in the future. There have been no randomised control trials performed comparing these three techniques and their risk of UES, this would be very beneficial in identifying which technique is truly superior. While open cystectomy is still the gold standard, and both robotic techniques are gaining in popularity, this should be feasible. We suggest further studies need to be performed in this area, paying particularly attention to the learning curve, to help improve our understanding of the benefits and risks of robotic surgery.

## Supplementary Information

Below is the link to the electronic supplementary material.

Supplementary file1 (XLSX 84 KB)

Supplementary file2 (XLSX 17 KB)

## Data Availability

Data is provided within the supplementary files.

## Abbreviations

BMI: Body mass index
CCI: Charleston co-morbidity index
DM: Daniel McNicholas
ECUD: Extracorporeal urinary diversion
ICG: Indo cyanine green
ICUD: Intracorporeal urinary diversion
IRCC: International robotic cystectomy consortium
NIRF: Near infrared fluorescence
OE: Omer El Taji
PRISMA: Preferred reporting items for systematic reviews and meta-analysis
RCT: Randomised control trial
UES: Uretero-enteric stricture
VH: Vishwanath Hanchanale
ZS: Zain Siddiqui

## References

[1] Witjes JA, Bruins HM, Cathomas R, Compérat EM, Cowan NC, Gakis G, Hernández V, Linares Espinós E, Lorch A, Neuzillet Y, Rouanne M, Thalmann GN, Veskimäe E, Ribal MJ, van der Heijden AG (2021) European association of urology guidelines on Muscle-invasive and metastatic bladder cancer: summary of the 2020 guidelines. Eur Urol 79(1):82–104. 10.1016/j.eururo.2020.03.055. (**Epub 2020 Apr 29 PMID: 32360052**) 32360052 10.1016/j.eururo.2020.03.055

[2] Bachour K, Faiena I, Salmasi A, Lenis AT, Johnson DC, Pooli A, Drakaki A, Pantuck AJ, Chamie K (2018) Trends in urinary diversion after radical cystectomy for urothelial carcinoma. World J Urol 36(3):409–416. 10.1007/s00345-017-2169-3. (**Epub 2018 Jan 3 PMID: 29299664**) 29299664 10.1007/s00345-017-2169-3

[3] Almassi N, Bochner BH (2020) Ileal conduit or orthotopic neobladder: selection and contemporary patterns of use. Curr Opin Urol 30(3):415–420. 10.1097/MOU.0000000000000738. (**PMID: 32141937; PMCID: PMC8261790**) 32141937 10.1097/MOU.0000000000000738 PMC8261790

[4] Menon M, Hemal AK, Tewari A, Shrivastava A, Shoma AM, El-Tabey NA, Shaaban A, Abol-Enein H, Ghoneim MA (2003) Nerve-sparing robot-assisted radical cystoprostatectomy and urinary diversion. Br J Urol Int 92(3):232–236. 10.1046/j.1464-410x.2003.04329.x. (**PMID: 12887473**) 10.1046/j.1464-410x.2003.04329.x 12887473

[5] Wang GJ, Barocas DA, Raman JD, Scherr DS (2008) Robotic vs open radical cystectomy: prospective comparison of perioperative outcomes and pathological measures of early oncological efficacy. Br J Urol Int 101(1):89–93. 10.1111/j.1464-410X.2007.07212.x. (**Epub 2007 Sep 20 PMID: 17888044**) 10.1111/j.1464-410X.2007.07212.x 17888044

[6] Galich A, Sterrett S, Nazemi T, Pohlman G, Smith L, Balaji KC (2006) Comparative analysis of early perioperative outcomes following radical cystectomy by either the robotic or open method. J Soc Laparosc Robot Surg 10(2):145–150 (**PMID: 16882409; PMCID: PMC3016134**) PMC3016134 16882409

[7] Rai BP, Bondad J, Vasdev N, Adshead J, Lane T, Ahmed K, Khan MS, Dasgupta P, Guru K, Chlosta PL, Aboumarzouk OM (2020) Robot-assisted vs open radical cystectomy for bladder cancer in adults. Br J Urol Int 125(6):765–779. 10.1111/bju.14870. (**PMID: 31309688**) 10.1111/bju.14870 31309688

[8] Goh AC, Belarmino A, Patel NA, Sun T, Sedrakyan A, Bochner BH, Hu JC (2020) A Population-based study of ureteroenteric strictures after open and robot-assisted radical cystectomy. Urology 135:57–65. 10.1016/j.urology.2019.07.054. (**Epub 2019 Oct 13 PMID: 31618656**) 31618656 10.1016/j.urology.2019.07.054

[9] Amin KA, Vertosick EA, Stearns G, Fathollahi A, Sjoberg DD, Donat MS, Herr H, Bochner B, Dalbagni G, Sandhu JS (2020) Predictors of benign ureteroenteric anastomotic strictures after radical cystectomy and urinary diversion. Urology 144:225–229. 10.1016/j.urology.2018.06.024. (**Epub 2018 Jun 30 PMID: 29964128; PMCID: PMC8672705**) 29964128 10.1016/j.urology.2018.06.024 PMC8672705

[10] Shimko MS, Tollefson MK, Umbreit EC, Farmer SA, Blute ML, Frank I (2011) Long-term complications of conduit urinary diversion. J Urol 185(2):562–567. 10.1016/j.juro.2010.09.096. (**Epub 2010 Dec 18 PMID: 21168867**) 21168867 10.1016/j.juro.2010.09.096

[11] Huang C, Assel M, Beech BB, Benfante NE, Sjoberg DD, Touijer A, Coleman JA, Dalbagni G, Herr HW, Donat SM, Laudone VP, Vickers AJ, Bochner BH, Goh AC (2022) Uretero-enteric stricture outcomes: secondary analysis of a randomised controlled trial comparing open versus robot-assisted radical cystectomy. Br J Urol Int 130(6):809–814. 10.1111/bju.15825. (**Epub 2022 Jun 25 PMID: 35694836**) 10.1111/bju.15825 PMC10454986 35694836

[12] Reesink DJ, Gerritsen SL, Kelder H, van Melick HHE, Stijns PEF (2021) Evaluation of ureteroenteric anastomotic strictures after the introduction of robot-assisted radical cystectomy with intracorporeal urinary diversion: results from a large tertiary referral center. J Urol 205(4):1119–1125. 10.1097/JU.0000000000001518. (**Epub 2020 Nov 30 PMID: 33249976**) 33249976 10.1097/JU.0000000000001518

[13] Ramahi YO, Shiekh M, Shah AA, Houenstein H, Ely HB, Shabir U, Jing Z, Li Q, Hussein AA, Guru KA (2023) Uretero-enteric Strictures after robot assisted radical cystectomy: prevalence and management over two decades. Clin Genitourin Cancer 21(2):e19–e26. 10.1016/j.clgc.2022.10.006. (**Epub 2022 Oct 12 PMID: 36372690**) 36372690 10.1016/j.clgc.2022.10.006

[14] Bricker EM (1950) Bladder substitution after pelvic evisceration. Surg Clin North Am 30(5):1511–1521. 10.1016/s0039-6109(16)33147-4. (**PMID: 14782163**) 14782163 10.1016/s0039-6109(16)33147-4

[15] Wallace DM (1966) Ureteric diversion using a conduit: a simplified technique. Br J Urol 38(5):522–527. 10.1111/j.1464-410x.1966.tb09747.x. (**PMID: 5332687**) 5332687 10.1111/j.1464-410x.1966.tb09747.x

[16] Davis NF, Burke JP, McDermott T, Flynn R, Manecksha RP, Thornhill JA (2015) Bricker versus Wallace anastomosis: a meta-analysis of ureteroenteric stricture rates after ileal conduit urinary diversion. Can Urol Assoc J 9(5–6):E284–E290. 10.5489/cuaj.2692. (**PMID: 26029296 PMCID: PMC4439225**) 26029296 10.5489/cuaj.2692 PMC4439225

[17] Kouba E, Sands M, Lentz A, Wallen E, Pruthi RS (2007) A comparison of the Bricker versus Wallace ureteroileal anastomosis in patients undergoing urinary diversion for bladder cancer. J Urol 178(3 Pt 1):945–948. 10.1016/j.juro.2007.05.030. (**Epub 2007 Jul 16 PMID: 17632159**) 17632159 10.1016/j.juro.2007.05.030

[18] Faraj KS, Rose KM, Navaratnam AK, Abdul-Muhsin HM, Eversman S, Singh V, Tyson MD (2021) Effect of intracorporeal urinary diversion on the incidence of benign ureteroenteric stricture after cystectomy. Int J Urol: Off J Japan Urol Assoc 28(5):593–597. 10.1111/iju.14521 10.1111/iju.14521 33594730

[19] Ericson KJ, Thomas LJ, Zhang JH, Knorr JM, Khanna A, Crane A, Zampini AM, Murthy PB, Berglund RK, Pascal-Haber G, Lee BHL (2020) Uretero-enteric anastomotic stricture following radical cystectomy: a comparison of open, robotic extracorporeal, and robotic intracorporeal approaches. Urology 144:130–135. 10.1016/j.urology.2020.06.047 32653565 10.1016/j.urology.2020.06.047

[20] López-Molina C, Carrion A, Campistol M, Piñero A, Lozano F, Salvador C, Raventós CX, Trilla E (2022) Evaluating the impact of the learning curve on the perioperative outcomes of robot-assisted radical cystectomy with intracorporeal urinary diversion. Actas Urológicas Españolas (English Ed) 46(1):57–62. 10.1016/j.acuroe.2021.05.004. (**Epub 2021 Nov 25 PMID: 34840098**) 10.1016/j.acuroe.2021.05.004 34840098

[21] Ahmed K, Khan SA, Hayn MH, Agarwal PK, Badani KK, Balbay MD, Castle EP, Dasgupta P, Ghavamian R, Guru KA, Hemal AK, Hollenbeck BK, Kibel AS, Menon M, Mottrie A, Nepple K, Pattaras JG, Peabody JO, Poulakis V, Pruthi RS, Redorta JP, Rha KH, Richstone L, Saar M, Scherr DS, Siemer S, Stoeckle M, Wallen EM, Weizer AZ, Wiklund P, Wilson T, Woods M, Khan MS (2014) Analysis of intracorporeal compared with extracorporeal urinary diversion after robot-assisted radical cystectomy: results from the International Robotic Cystectomy Consortium. Eur Urol 65(2):340–347. 10.1016/j.eururo.2013.09.042. (**Epub 2013 Oct 9 PMID: 24183419**) 24183419 10.1016/j.eururo.2013.09.042

[22] Wilson TG, Guru K, Rosen RC, Wiklund P, Annerstedt M, Bochner BH, Chan KG, Montorsi F, Mottrie A, Murphy D, Novara G, Peabody JO, Palou Redorta J, Skinner EC, Thalmann G, Stenzl A, Yuh B, Catto J (2015) Best practices in robot-assisted radical cystectomy and urinary reconstruction: recommendations of the Pasadena Consensus Panel. Eur Urol 67(3):363–375. 10.1016/j.eururo.2014.12.009. (**Epub 2015 Jan 9 PMID: 25582930**) 25582930 10.1016/j.eururo.2014.12.009

[23] Page MJ, McKenzie JE, Bossuyt PM, Boutron I, Hoffmann TC, Mulrow CD, Shamseer L, Tetzlaff JM, Akl EA, Brennan SE, Chou R, Glanville J, Grimshaw JM, Hróbjartsson A, Lalu MM, Li T, Loder EW, Mayo-Wilson E, McDonald S, McGuinness LA, Stewart LA, Thomas J, Tricco AC, Welch VA, Whiting P, Moher D (2021) The PRISMA 2020 statement: an updated guideline for reporting systematic reviews. BMJ 29(372):n71. 10.1136/bmj.n71. (**PMID:33782057; PMCID:PMC8005924**) 10.1136/bmj.n71 PMC8005924 33782057

[24] Wells GASB, O’Connell D, Peterson J, et al. The Newcastle -Ottawa scale (NOS) for assessing the quality if nonrandomized studies in meta-analyses. 2011, http://www.ohri.ca/programs/clinical_epidemiology/oxford.asp. Accessed 24 Jan 2024.

[25] Lone Z, Murthy PB, Zhang JH, Ericson KJ, Thomas L, Khanna A, Haber GP, Lee BH (2021) Comparison of renal function after open radical cystectomy, extracorporeal robot assisted radical cystectomy, and intracorporeal robot assisted radical cystectomy. Urol Oncol 39(5):301.e1-301.e9. 10.1016/j.urolonc.2020.09.018. (**Epub 2020 Oct 6 PMID: 33036904**) 33036904 10.1016/j.urolonc.2020.09.018

[26] Parekh DJ, Reis IM, Castle EP, Gonzalgo ML, Woods ME, Svatek RS, Weizer AZ, Konety BR, Tollefson M, Krupski TL, Smith ND, Shabsigh A, Barocas DA, Quek ML, Dash A, Kibel AS, Shemanski L, Pruthi RS, Montgomery JS, Weight CJ, Thompson IM (2018) Robot-assisted radical cystectomy versus open radical cystectomy in patients with bladder cancer (RAZOR): an open-label, randomised, phase 3, non-inferiority trial. Lancet (London, England) 391(10139):2525–2536. 10.1016/S0140-6736(18)30996-6 29976469 10.1016/S0140-6736(18)30996-6

[27] Ahmed YE, Hussein AA, May PR, Ahmad B, Ali T, Durrani A, Khan S, Kumar P, Guru KA (2017) Natural history, predictors and management of ureteroenteric strictures after robot assisted radical cystectomy. J Urol 198(3):567–574. 10.1016/j.juro.2017.02.3339. (**Epub 2017 Mar 1 PMID: 28257782**) 28257782 10.1016/j.juro.2017.02.3339

[28] Anderson CB, Morgan TM, Kappa S, Moore D, Clark PE, Davis R, Penson DF, Barocas DA, Smith JA Jr, Cookson MS, Chang SS (2013) Ureteroenteric anastomotic strictures after radical cystectomy-does operative approach matter? J Urol 189(2):541–547. 10.1016/j.juro.2012.09.034. (**Epub 2012 Dec 20 PMID: 23260561**) 23260561 10.1016/j.juro.2012.09.034

[29] EAU Guidelines. Edn. Presented at the EAU Annual Congress Milan 2023. Section 8.3. ISBN 978-94-92671-19-6. https://uroweb.org/guidelines/muscle-invasive-and-metastatic-bladder-cancer/chapter/followup. Accessed 11 Sep 2023.

[30] Clark PB (1979) End-to-end ureteroileal anastomosis for ileal conduits. Br J Urol 51(2):105–109. 10.1111/j.1464-410x.1979.tb02841.x. (**PMID: 465967**) 465967 10.1111/j.1464-410x.1979.tb02841.x

[31] Shah SH, Movassaghi K, Skinner D, Dalag L, Miranda G, Cai J, Schuckman A, Daneshmand S, Djaladat H (2015) Ureteroenteric strictures after open radical cystectomy and urinary diversion: the university of southern california experience. Urology 86(1):87–91. 10.1016/j.urology.2015.03.014. (**Epub 2015 May 16 PMID: 25987494**) 25987494 10.1016/j.urology.2015.03.014

[32] Chan KG, Collins JW, Wiklund NP (2015) Robot-assisted radical cystectomy: extracorporeal vs intracorporeal urinary diversion. J Urol 193(5):1467–1469. 10.1016/j.juro.2015.02.042. (**Epub 2015 Feb 14 PMID: 25686541**) 25686541 10.1016/j.juro.2015.02.042

[33] Ahmadi N, Ashrafi AN, Hartman N, Shakir A, Cacciamani GE, Freitas D, Rajarubendra N, Fay C, Berger A, Desai MM, Gill IS, Aron M (2019) Use of indocyanine green to minimise uretero-enteric strictures after robotic radical cystectomy. Br J Urol Int 124(2):302–307. 10.1111/bju.14733. (**Epub 2019 Apr 11 PMID: 30815976**) 10.1111/bju.14733 30815976

[34] Shen JK, Jamnagerwalla J, Yuh BE, Bassett MR, Chenam A, Warner JN, Zhumkhawala A, Yamzon JL, Whelan C, Ruel NH, Lau CS, Chan KG (2019) Real-time indocyanine green angiography with the SPY fluorescence imaging platform decreases benign ureteroenteric strictures in urinary diversions performed during radical cystectomy. Ther Adv Urol 11:1756287219839631. 10.1177/1756287219839631 31057669 10.1177/1756287219839631 PMC6452578

